# The International Society for Computational Biology and WikiProject Computational Biology: celebrating 10 years of collaboration towards open access

**DOI:** 10.1093/bioinformatics/btx388

**Published:** 2017-06-19

**Authors:** Kieran O’Neill, Vivek Rai, Alastair M Kilpatrick

**Affiliations:** 1 Department of Pathology and Laboratory Medicine, Faculty of Medicine, University of British Columbia, BC, Canada; 2 Michael Smith Genome Sciences Centre, BC Cancer Agency, Vancouver, BC, Canada; 3 Department of Biotechnology, Indian Institute of Technology, Kharagpur, India; 4 MRC Centre for Regenerative Medicine, University of Edinburgh, Edinburgh, UK

Open access to scientific information is a core principle of the International Society for Computational Biology (ISCB). This principle is shared by the Wikimedia Foundation, with its primary goal to collect, develop and disseminate free and open-access educational content. Consequently, ISCB has and continues to foster strong links with several Wikimedia projects, particularly Wikipedia.

To this end, ISCB works closely with WikiProject Computational Biology (WCB), a group of around 130 editors overseeing Wikipedia articles relating to computational biology and bioinformatics. In 2017, WCB celebrates its 10th anniversary, having grown to cover more than 1,300 articles in the English Wikipedia. This article serves to acknowledge past ISCB–WCB collaborations, release the results of the 2016–2017 ISCB Wikipedia competition, officially announce the 2017–2018 competition, and explore exciting future directions, including the potential role of WCB in classroom education for computational biology.

## 1 Topic pages, competitions and editathons: a history of collaboration

WCB’s 10-year history has been marked by a number of collaborative efforts with ISCB. These include PLOS Topic Pages, ISCB’s annual Wikipedia competition, and events at ISCB conferences.

Topic Pages are an initiative pioneered in 2012 by *PLOS Computational Biology*, one of the official journals of ISCB, to create high-quality Wikipedia articles while also providing trainees with an opportunity to build a publication record. A topic page is a review article written for the general public and according to Wikipedia guidelines that is simultaneously published by PLOS and as a Wikipedia article. Since its inception, 10 high quality, in-depth articles on topics in computational biology have been created.

Also since 2012, ISCB and WCB have held an annual Wikipedia competition, offering cash prizes and memberships to students and trainees for the best contributions to computational biology-related Wikipedia articles and Wikidata entries (Bateman *et al.*, 2013; Kilpatrick, 2016). This competition has been highly successful in driving the improvement of coverage of computational biology within Wikipedia, having drawn a total of 144 entries over just five years.

Beyond the ISCB Wikipedia Competition and PLOS Topic Pages, the partnership between the ISCB and WCB has continued to grow. Following tutorials at ECCB 2012 and ISMB 2014 which provided a practical introduction to editing Wikipedia for scientists, the ISCB held a Wikipedia and Wikidata editathon at ISMB 2016 as part of the celebrations for the Wikipedia Year of Science.

Another editathon will be held at ISMB/ECCB 2017 on an all-day drop-in model, and we invite attendees regardless of their previous experience with Wikipedia editing. We envisage similar joint events in the future which will help to improve the coverage of computational biology and bioinformatics on Wikimedia projects.

## 2 Results of the 2016–2017 ISCB Wikipedia competition

There were 17 substantial entries into the 2016–2017 Wikipedia competition, including five entries for non-English articles. A panel of six ISCB judges considered these articles, as in previous competitions, based on the clarity of the writing, depth of knowledge of the subject, and quality of figures and images used. The judging panel selected the following as the winners of the 2016–2017 ISCB Wikipedia competition:

First prize: Yadi Zhou (Ohio University, USA) for improvements to the Smith-Waterman algorithm article, in both English and Chinese Wikipedia.

Second prize: Charlotte Klimovich (Ohio University, USA) for improvements to the Margaret Oakley Dayhoff article.

Third prize: Nicole Wheeler (University of Canterbury, New Zealand) for improvements to the Pfam article.

## 3 The 2017–2018 ISCB Wikipedia competition

During the 2017–2018 academic year, ISCB is running the 6th annual ISCB Wikipedia Competition in collaboration with WCB. Entry to the competition is open internationally to students and trainees of any level, both as individuals and as groups. As in previous years, the ISCB encourages competition entries for contributions to Wikipedia in any language, and contributions to Wikidata items. Further information about the competition can be found here: https://en.wikipedia.org/wiki/WP:ISCB2017

The competition will begin on 21 July 2017, coinciding with the ISMB/ECCB 2017 conference, and will end on 31 December 2017. Each article entered in the competition will be reviewed by students nominated by the ISCB Student Council, and a shortlist of entries will be examined by a judging panel. The winners will be presented with their awards at ISMB 2018 in Chicago.

## 4 Strengthening links between Wikimedia and ISCB: Wikipedia in the (Bioinformatics) classroom

The ISCB also sees a role for WCB in collaborating with the ISCB Education Committee which, as part of its remit, seeks to define curriculum guidelines for bioinformatics courses (Welch *et al.*, 2016). One potential area of work may be to use the current draft guidelines in order to identify a subset of Wikipedia articles which would provide a ‘core’ set of articles for a bioinformatics curriculum. Clearly these articles should be as complete as possible and should be prioritized for improvement by WCB members. The ISCB suggests that Wikipedia also has scope for helping to refine the curriculum guidelines. As an example, Wikipedia keeps viewing statistics for each of its articles; these show that the Bioinformatics article page was viewed over 41 000 times in January 2017 alone and the most-viewed WCB article (on CRISPR) was viewed almost 140 000 times in the same period. These viewing statistics may play a useful role in identifying areas of the curriculum that are of particular importance and therefore should be emphasized in future versions of the curriculum.

We also encourage teachers, tutors and lecturers at all levels to use the ISCB Wikipedia competition as part of their class assignments. For example, by having students ‘claim’ a Wikipedia article for the competition, with their contributions over a short, defined period in the fall semester marked by the instructor. Wikipedia provides information and resources for running editing projects as part of classroom activities and the “Ten Simple Rules for Editing Wikipedia” (Logan *et al.*, 2010) also provides an excellent resource for those new to Wikipedia editing.

Besides the competition, there are a number of ways in which Wikipedia may be used in a classroom setting. For example, both undergraduates and graduate students may ‘claim’ an article to improve as a short class project to be evaluated by the tutor. Incentives such as the prospect of appearing on the front page of Wikipedia as a ‘Did you know?’ article can help to provide extra motivation. For graduate students, a Wikipedia article can potentially be expanded into a PLOS Topic Page (Wodak *et al.*, 2012), which offers an opportunity to build a publication record, and can grant a head start on writing a thesis introduction. As noted previously, the collaborative writing environment of Wikipedia in particular encourages critical thinking and improves research skills.

## 5 Conclusions

**Fig. 1:**
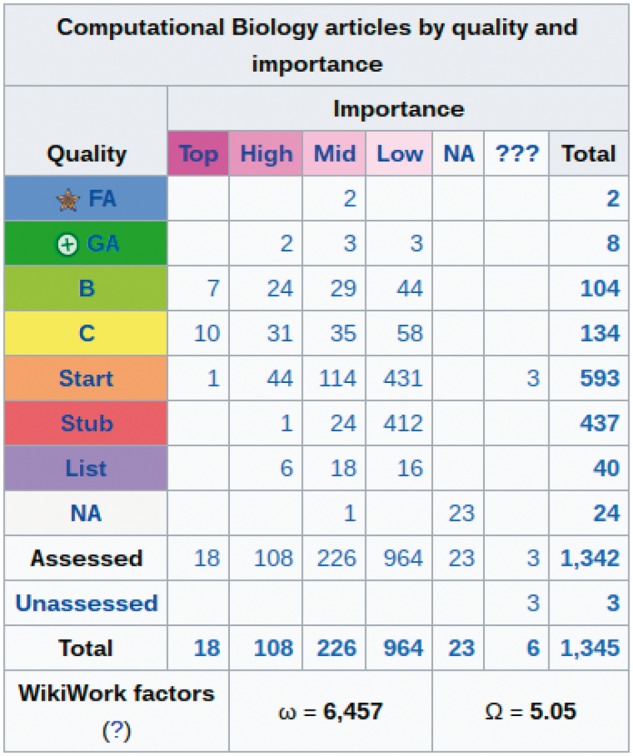
Wikipedia computational biology articles rated for both quality and importance by WCB. Quality levels are ordered from highest to lowest, beginning with ‘Featured Articles’ (considered to be amongst the best articles in Wikipedia) and ‘Good Articles’ (articles which meet a core set of Wikipedia editorial standards but are not FA quality). List articles are not rated for quality (Color version of this figure is available at *Bioinformatics* online.)

ISCB and WCB have spent the last decade working together to improve access to information about computational biology by fostering the creation of high-quality Wikipedia articles. The results of this have been encouraging. A paper in 2013 (Bateman *et al.*, 2013) reported on the state of articles within the scope of WCB. Since then, almost 200 new articles have been added and many of the shortest articles (known as stubs) have been expanded (Fig. 1). However, a significant number of articles still remain at ‘Start class’ or lower, indicating weaknesses in many areas. ISCB and WCB are keen to expand on the depth and quality of articles relating to computational biology and encourage students and trainees at all levels to take part in the ISCB Wikipedia Competition, write topic pages, and drop in to the Wikipedia Editathon at ISMB/ECCB 2017. We look forward to continued collaborations and efforts by the entire community towards enhancing and disseminating humanity’s knowledge of computational biology.

*Conflict of interest:* none declared.
